# Antidepressant side effects and their impact on treatment outcome in people with major depressive disorder: an iSPOT-D report

**DOI:** 10.1038/s41398-021-01533-1

**Published:** 2021-08-04

**Authors:** Taylor A. Braund, Gabriel Tillman, Donna M. Palmer, Evian Gordon, A. John Rush, Anthony W. F. Harris

**Affiliations:** 1 Brain Dynamics Centre, The Westmead Institute for Medical Research, Sydney, NSW Australia; 2 Discipline of Psychiatry, Sydney Medical School, University of Sydney, Sydney, NSW Australia; 3 School of Science, Psychology and Sport, Federation University, Ballarat, VIC Australia; 4 Total Brain, Sydney, NSW Australia; 5 Total Brain, San Francisco, CA USA; 6 Department of Psychiatry & Behavioral Sciences, Duke University, School of Medicine, Durham, NC USA; 7 Department of Psychiatry, Texas Tech University Health Sciences Center, Lubbock, TX USA; 8 Professor Emeritus, Duke-National University of Singapore, Singapore, Singapore

**Keywords:** Predictive markers, Physiology, Human behaviour

## Abstract

Side effects to antidepressant medications are common and can impact the prognosis of successful treatment outcome in people with major depressive disorder (MDD). However, few studies have investigated the severity of side effects over the course of treatment and their association with treatment outcome. Here we assessed the severity of side effects and the impact of treatment type and anxiety symptoms over the course of treatment, as well as whether side effects were associated with treatment outcome. Participants were *N* = 1008 adults with a current diagnosis of single-episode or recurrent, nonpsychotic MDD. Participants were randomised to receive escitalopram, sertraline, or venlafaxine-extended release with equal probability and reassessed at 8 weeks regarding Hamilton Rating Scale Depression (HRSD_17_) and Quick Inventory of Depressive Symptomatology (QIDS-SR_16_) remission and response. Severity of side effects were assessed using the Frequency, Intensity, and Burden of Side Effects Rating (FIBSER) scale and assessed at day 4 and weeks 2, 4, 6, and 8. Frequency, intensity, and burden of side effects were greatest at week 2, then only frequency and intensity of side effects gradually decreased up to week 6. Treatment type and anxiety symptoms did not impact the severity of side effects. A greater burden—but not frequency or intensity—of side effects was associated with poorer treatment outcome and as early as 4 days post-treatment. Together, this work provides an informative mapping of the progression of side effects throughout the treatment course and their association with treatment outcome. Importantly, the burden of side effects that are present as early as 4 days post-treatment predicts poorer treatment outcome and should be monitored closely. iSPOT-D: Registry name: ClinicalTrials.gov. Registration number: NCT00693849.

## Introduction

Selective serotonin reuptake inhibitors (SSRIs) and serotonin–norepinephrine reuptake inhibitors (SNRIs) are first-line pharmacological treatments for people with major depressive disorder (MDD) [1] and anxiety disorders [2]. However, SSRIs and SNRIs are associated with a range of side effects, including loss of appetite, weight loss, drowsiness, dizziness, fatigue, headaches, increased suicidal thoughts, nausea/vomiting, sexual dysfunction, and increased risk of cardiovascular and cerebrovascular events [3–5]. While specific antidepressant side effects are diverse, they also occur generally with a high frequency, intensity, and burden [6, 7]. Previous studies suggest that frequency, intensity, and burden of side effects are impacted by antidepressant treatment type, anxiety symptoms, and the presence of anxious depression (for a review, see Ionescu et al. [8]).

Anxious depression is present in approximately half of the MDD population and is commonly defined either syndromally (i.e., co-occurring MDD and anxiety disorder diagnoses) or using 17-item Hamilton Rating Scale Depression (HRSD_17_) criteria (i.e., MDD diagnosis and an HRSD_17_ anxiety/somatisation factor score of ≥7 [9–11]). Some, but not all studies suggest that anxious depression increases the severity of side effects (for a review, see Ionescu et al. [8]). For example, people with anxious depression were found to have a greater maximum frequency, intensity, and burden of side effects compared to people with non-anxious depression in the Sequenced Treatment Alternatives to Relieve Depression (STAR*D) trial [12]. A greater side effect severity has also been found at various study endpoints in people with anxious depression compared to people with MDD only [13–15]. However, these associations are not found in all definitions of anxious depression, with Gaspersz et al. [14]. finding no differences in the frequency of side effects between people with syndromal and non-syndromal anxious depression.

Few studies have assessed how side effects progress throughout treatment and their association with treatment outcome [8]. Given that the presence of anxiety symptoms and treatment type may impact side effect profiles [8], the aim of the current study was to assess the severity of side effects—as well as the impact of anxiety symptoms and treatment type—over the course of treatment in a large patient sample from the international Study to Predict Optimized Treatment for Depression (iSPOT-D) [16]. We also tested whether early ratings of side effects predicted treatment outcome. We expected that, in line with previous studies [8], treatment type and anxiety symptoms would impact the frequency, intensity, and burden of side effects. We also expected that higher side effect ratings would predict poorer treatment outcome.

## Methods

### Study overview

The iSPOT-D is a phase-IV, multi-site, international, randomized, open-label trial designed to identify markers of treatment response to commonly prescribed medications in an adult depressed, outpatient population. All participants were either antidepressant medication naive or washed out. Participants were randomized to receive escitalopram, sertraline, or venlafaxine-extended release (venlafaxine-XR) with equal probability. Assessments were collected at pre-treatment and post-treatment at 8 weeks. Study site personnel contacted participants by telephone at day 4 and weeks 2, 4, and 6 to monitor antidepressant dosage, compliance, concomitant medications, and adverse events. The iSPOT-D trial was designed with no placebo arm and participants were aware of the medication that they were taking to best match real-world practice. In this way, findings also reflect treatment regimens that exist in routine practice and promote the translatability of the findings. Data were collected between December 1, 2008 and September 30, 2013. For more details on the study protocol design, rationale and methods, see Williams et al. [16].

### Participants

Participants (*N* = 1008) were adults (age 18–65 years) with a current diagnosis of single-episode or recurrent, nonpsychotic MDD (CONSORT chart provided in Supplementary Fig. 1). Participants were diagnosed on the Mini-International Neuropsychiatric Interview—Plus (MINI-Plus) [17] according to Diagnostic and Statistical Manual of Mental Disorders (DSM)-IV criteria [18]. All participants required a HRSD_17_ score >16 at entry. For a full list of inclusion and exclusion criteria, see Supplementary Fig. 2. Participants provided written informed consent after receiving a complete description of the study. The study was approved by institutional or ethical review boards at each site, and its protocols followed International Conference on Harmonization and Good Clinical Practice principles, the U.S. Food and Drug Administration Code of Federal Regulations, and country-specific guidelines (see Supplementary Table 1 for individual iSPOT-D study management sites and investigators).

### Depression severity

Depression severity was assessed using the HRSD_17_ [19] and the 16-Item Quick Inventory of Depressive Symptomatology-Self-Rated (QIDS-SR_16_) [20, 21]. The HRSD_17_ is a 17-item clinician-rated scale scored either on a 3-point or 5-point Likert scale used to rate severity of their depression by gauging mood, feelings of guilt, suicide ideation, insomnia, agitation or retardation, anxiety, weight loss, and somatic symptoms [19]. The QIDS-SR_16_ is a 16-item questionnaire scored on a 4-point Likert scale used to rate the severity of depression by assessing the severity of the nine DSM-IV diagnostic symptom domains for MDD. Remission was defined as a week 8 HRSD_17_ score ≤7 or a week 8 QIDS-SR_16_ score ≤5 [20, 21]. Response was defined as a ≥50% decrease from baseline on the HRSD_17_ or QIDS-SR_16_. In line with the study protocol, the primary outcomes were rates of remission and response on the HRSD_17_. The secondary outcome was remission and response on the QIDS-SR_16_. The HRSD_17_ has shown good internal consistency (*α* = 79) and test–retest reliability (*r* = 0.87) in a recent meta-analysis [22]. The QIDS-SR_16_ has also shown good internal consistency (*α* = 0.86), as well as convergent and discriminant validity [20, 23].

### Frequency, intensity, and burden of side effects

Side effect severity was measured using the self-reported Frequency, Intensity, and Burden of Side Effects Rating (FIBSER) [24] scale. The FIBSER assesses three domains of antidepressant medication side effect impact, including Frequency (frequency of side effects of medications taken within the past week for depression), Intensity (intensity of side effects due to medications taken within the last week for depression), and Burden (degree to which antidepressant medication side effects over the last week interfered with day-to-day functions). Frequency, Intensity, and Burden was rated on a 7-point scale, ranging from “no side effects” to “present all the time” for frequency, from “no side effects” to “intolerable” for intensity, and from “no impairment” to “unable to function due to side effects” for burden (see Supplementary Table 2 for categorical distribution of FIBSER scores at each study timepoint).

### Adverse events

Adverse events were recorded and coded using the Medical Dictionary for Regulatory Activities (MedDRA) [25]. MedDRA is a multi-axial, five-tiered hierarchical terminology used by regulatory authorities and the biopharmaceutical industry for the coding and classification of adverse events. Adverse events were reported using their highest hierarchical grouping (i.e., system organ classes). Adverse events were determined as related to the study treatments (i.e., potential side effect to study treatment) by the investigator and in line with the study protocol. The terminology was developed and is endorsed by the International Conference on Harmonization of Technical Requirements for Registration of Pharmaceuticals for Human Use.

### Anxious depression definitions

#### Syndromal anxious depression

Syndromal anxious depression was defined as a DSM-IV MDD diagnosis and at least one concurrent MINI-Plus identified anxiety disorder, including generalized anxiety disorder, panic disorder, agoraphobia, social phobia, and specific phobia [9].

#### HRSD_17_ anxious depression

HRSD_17_ anxious depression was defined as a DSM-IV MDD diagnosis and a HRSD_17_ anxiety/somatization factor score of ≥7 [9]. The anxiety/somatisation factor was derived from a factor analyses of the HRSD_17_ by Cleary and Guy [26] and includes six items: hypochondriasis, insight, general and gastrointestinal somatic symptoms, and psychic and somatic anxiety.

### Depression, anxiety, and stress scale

Anxiety was also assessed using the self-rated 42-item Depression Anxiety and Stress Scale (DASS_42_) [27, 28]. DASS_42_ item scores range from 0 (did not apply to me at all) to 3 (applied to me very much or most of the time). The 14-item DASS_42_ anxiety subscale, used in this study, includes 14 items measuring autonomic arousal, skeletal muscle effects, situational anxiety, and subjective experience of anxious affect.

### Protocol treatment

Participants were randomized to receive escitalopram, sertraline, or venlafaxine-XR with equal probability. All psychotropic medications (except sleep aids and anxiolytics) were discontinued and washed out prior to baseline assessments. Antidepressants were prescribed and doses were adjusted by the participant’s treating physician according to routine clinical practice. Additional medication for associated symptoms (e.g., insomnia) or medication-induced side effects (e.g., nausea) were allowed as they reflect common practice. Any treatment for concurrent general medical conditions, except medications contraindicated with the study-assigned antidepressants, were allowed and recorded.

### Statistical analysis

To test whether timepoint, treatment type (i.e., individual treatment arm), DASS_42_ anxiety subscale, HRSD_17_ anxiety/somatization, and presence of anxious depression were associated with side effects, we used hierarchical linear mixed effect models. Mixed effect models are well suited to the data as they retain participants with missing data and allow for the post hoc tests. We used a model comparison approach with the Akaike Information Criterion (AIC) [29] and Bayesian Information Criterion (BIC) [30], which selects the best fitting model while penalizing for complexity (i.e., number of parameters in the model). All models included side effect scores as the dependent variable, included subjects as a random effect, and included the fixed effect covariates of age, sex, baseline depression severity (measured via the QIDS-SR_16_), dosage at study outcome, and age of MDD onset. We then tested a set of models that either added the fixed effect variable timepoint or the fixed effect variables timepoint + treatment type, timepoint + DASS_42_ anxiety, timepoint + HRSD_17_ anxiety/somatization, or timepoint + anxious depression. Continuous fixed effects of age, depression severity, DASS_42_ anxiety, HRSD_17_ anxiety/somatization, age of MDD onset, and treatment dosage were grand mean centered. Post hoc *t* tests were performed using estimated marginal means and corrected for using the Bonferroni–Holm [31] method.

Pearson correlations were used to test for associations between severity of side effects and the QIDS-SR_16_ total score at each study timepoint. Logistic regression models were used to test whether the severity of side effects at each study timepoint were associated with antidepressant treatment outcome. Logistic regression models were also adjusted for the same covariates as those used in the hierarchical linear mixed effect models to test whether side effects were associated with treatment outcome independent of these covariates.

All analyses were conducted using R 3.5.1 [32]. Mixed linear models were tested using the “lme4” package in R [33]. *p* Values for mixed linear models were calculated using the “lmerTest” package in R [34]. Post hoc tests were performed using the “emmeans” package in R [35].

## Results

### Demographics

iSPOT-D demographics have been published elsewhere [6]. Briefly, there were 571 females (56.6%), with a mean age of 37.9 (SD = 12.6), 14.5 (SD = 2.8) years of education, 22.28 (SD = 12.0) age of MDD onset, and a QIDS-SR_16_ baseline score of 14.5 (SD = 3.8). Syndromal anxious depression was present in 261/1008 (25.9%) and HRSD_17_ anxious depression was present in 422/1008 (41.9%), with a mean HRSD_17_ anxiety/somatisation score of 6.16 (SD = 1.90; see Supplementary Fig. 3 for distribution of HRSD_17_ anxiety/somatisation factor scores). Mean dosages at week 8 were 12.5 mg/day (SD = 9.3) for escitalopram, 59.6 mg/day (SD = 33.2) for sertraline, and 76.1 mg/day (SD = 41.5) for venlafaxine-XR. Forty-two participants dropped out due to safety, tolerability, or efficacy reasons (see Supplementary Table 3 for distribution of last recorded FIBSER scores).

### Frequency, intensity, and burden of side effects

Table 1 shows the model comparison results for the hierarchical linear mixed effect models. The best fitting models overall (i.e., “the timepoint models”) included subjects as a random effect, the fixed effect timepoint, as well as the fixed effect covariates of age, sex baseline depression severity, dosage at study outcome, and age of MDD onset (frequency timepoint model: AIC = 9970.9, BIC = 10042.6; *χ*^2^(4) = 61.77, *p* < 001, intensity timepoint model: AIC = 9393.1, BIC = 9464.8; *χ*^2^(4) = 93.99, *p* < 001, burden timepoint model: AIC = 8409.0, BIC = 8480.7; *χ*^2^(4) = 57.96, *p* < 001). However, including treatment type, DASS_42_ anxiety, HRSD_17_ anxiety/somatization, or the presence of anxious depression did not improve the model’s fit to the data (for significant covariates, see Supplementary Material). Results did not change when removing those who dropped out due to safety, tolerability, or efficacy reasons (see Supplementary Table 4).

**Table 1:** Results from hierarchical mixed effect models comparing covariate only models to those including timepoint and those including timepoint to those including treatment type, anxious symptom severity, and presence of anxious depression.

Models	AIC	FIBSER—Frequency				AIC	FIBSER—Intensity				AIC	FIBSER—Burden			
		BIC	*χ*^2^	df	*p*		BIC	*χ*^2^	df	*p*		BIC	*χ*^2^	df	*p*
Covariate model	10,024.7	10,072.5	—	—	—	9479.1	9526.8	—	—	—	8459.0	8506.7	—	—	—
+Timepoint	9970.9	10,042.6	61.77	4	<0.001	9393.1	9464.8	93.99	4	<0.001	8409.0	8480.7	57.96	4	<0.001
+Timepoint + treatment	9971.3	10,054.9	3.64	2	0.162	9392.3	9475.9	4.79	2	0.091	8409.2	8492.8	3.80	2	0.149
+Timepoint + DASS_42_ anxiety	9972.7	10,050.4	0.20	1	0.652	9395.0	9472.7	0.05	1	0.826	8410.7	8488.3	0.35	1	0.556
+Timepoint + HRSD_17_ anxiety/somatization	9972.8	10,050.4	0.18	1	0.667	9394.6	9472.3	0.44	1	0.510	8409.0	8486.7	2.00	1	0.157
+Timepoint + HRSD_17_ anxious depression	9971.2	10,048.8	1.77	1	0.184	9394.7	9472.4	0.32	1	0.570	8410.9	8488.6	0.10	1	0.750
+Timepoint + syndromal anxious depression	9972.9	10,050.6	0.01	1	0.931	9394.6	9472.3	0.43	1	0.513	8410.9	8488.5	0.11	1	0.735

*FIBSER* Frequency, Intensity, and Burden of Side Effects Rating, *AIC* Akaike Information Criterion, *BIC* Bayesian Information Criterion.

We also tested whether people with anxious depression had a greater maximum frequency, intensity and burden of side effects compared to people with non-anxious depression. However, there were no differences in the maximum frequency, intensity, or burden of side effects in people with HRSD or syndromal anxious depression compared to non-anxious depression (see Supplementary Table 5).

### Post hoc tests

**Fig. 1:**
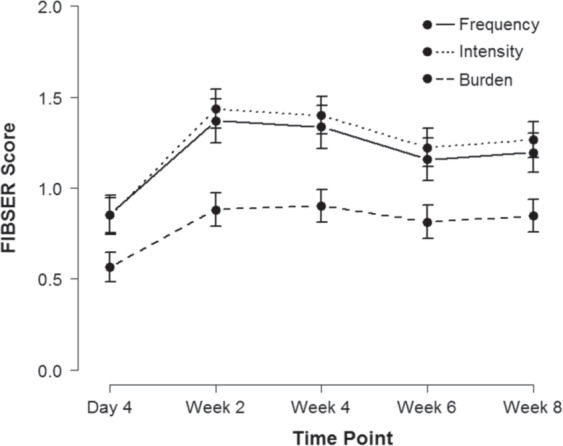
Mean FIBSER score trajectories. Note: Error bars represent 95% confidence intervals of the mean.

Figure 1 shows the mean FIBSER score trajectories (for all post hoc test results, see Supplementary Table 6). Post hoc tests revealed that, compared to day 4, there was a greater frequency, intensity, and burden of side effects at weeks 2, 4, 6, and 8 (all *p* < 0.001). Frequency and intensity of side effects at week 2 were greater compared to weeks 6 (both *p* = 0.002) and 8 (*p* = 0.012 and 0.009, respectively), but not week 4. Frequency and intensity of side effects at week 4 were greater compared to weeks 6 (*p* = 0.012 and 0.006, respectively), but only intensity was greater compared to week 8 (*p* = 0.020). There were no differences in frequency, intensity, and burden of side effects between weeks 6 and 8.

### Correlations between QIDS-SR_16_ and side effects

Table 2 shows the correlations between QIDS-SR_16_ scores and FIBSER scores at each study timepoint. Only burden of side effects was significantly but weakly correlated with QIDS-SR_16_ scores at day 4. Following day 4, FIBSER scores showed significant weak positive correlations with QIDS-SR_16_ at each study timepoint. The strength of correlations between the FIBSER and QIDS-SR_16_ at each study timepoint increased as the study progressed up to week 6, then decreased at week 8.

**Table 2:** Correlations between QIDS-SR_16_ scores and FIBSER scores at each study timepoint.

Timepoint	QIDS-SR_16_	FIBSER—Frequency				FIBSER—Intensity				FIBSER—Burden			
	*M* (SD)	*M* (SD)	*N*	*r*	*p*	*M* (SD)	*N*	*r*	*p*	*M* (SD)	*N*	*r*	*p*
Day 4	12.78 (4.27)	0.85 (1.46)	715	0.03	0.373	0.85 (1.32)	714	0.01	0.892	0.56 (1.11)	715	0.08	0.033
Week 2	10.4 (4.57)	1.37 (1.63)	699	0.16	<0.001	1.43 (1.46)	699	0.17	<0.001	0.88 (1.25)	699	0.23	<0.001
Week 4	9.20 (4.72)	1.34 (1.56)	673	0.24	<0.001	1.40 (1.38)	673	0.27	<0.001	0.90 (1.20)	673	0.33	<0.001
Week 6	8.48 (4.88)	1.16 (1.49)	619	0.35	<0.001	1.22 (1.33)	618	0.38	<0.001	0.81 (1.18)	619	0.39	<0.001
Week 8	7.63 (4.72)	1.19 (1.44)	688	0.27	<0.001	1.27 (1.32)	687	0.29	<0.001	0.85 (1.20)	688	0.34	<0.001

*FIBSER* Frequency, Intensity, and Burden of Side Effects, *QIDS-SR*_*16*_ 16-item Quick Inventory of Depression Symptomatology—Self Reported.

### Side effects predicting antidepressant treatment outcome

**Fig. 2:**
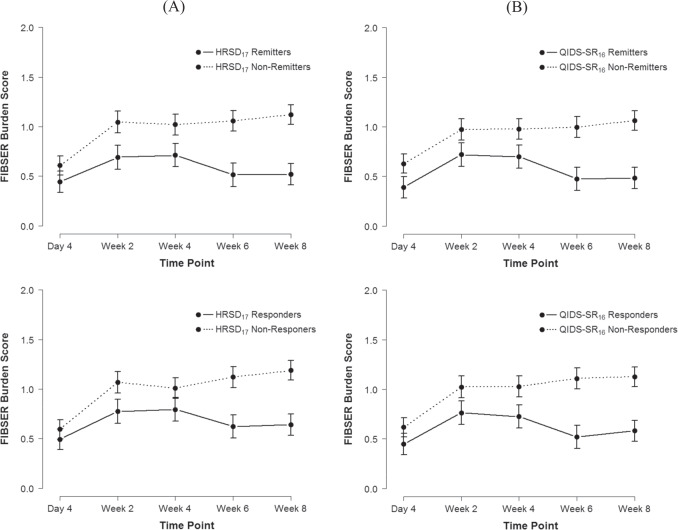
Mean FIBSER burden score trajectories for remitters and responders. **A** HRSD_17_ and **B** QIDS-SR_16_ remitters and responders. Note: Error bars represent 95% confidence intervals of the mean.

Table 3 shows the results from logistic regression models testing whether frequency, intensity, and burden of side effects predicted HRSD_17_ and QIDS-SR_16_ remission and response after adjusting for covariates age, sex, baseline depression severity (measured via the QIDS-SR_16_), dosage at study outcome, and age of MDD onset. Figure 2 shows the side effect trajectories of HRSD_17_ and QIDS-SR_16_ remitters and responders. A greater burden of side effects at week 2 was associated with poorer HRSD_17_ remission, and greater intensity of side effects at week 6 was associated with poorer HRSD_17_ remission. A greater burden of side effects was associated with poorer QIDS-SR_16_ remission and response at every timepoint, except for QIDS-SR_16_ response at week 4. A greater intensity of side effects at week 2 was also associated with poorer remission.

**Table 3:** Results from logistic regression models testing whether frequency, intensity, and burden of side effects predicted QIDS-SR_16_ and HRSD_17_ remission and response after adjusting for covariates.

Timepoint/measure		HRSD_17_ remission			HRSD_17_ response			QIDS-SR_16_ remission			QIDS-SR_16_ response		
		OR	CI 95% (lower, upper)	*p*	OR	CI 95% (lower, upper)	*p*	OR	CI 95% (lower, upper)	*p*	OR	CI 95% (lower, upper)	*p*
Day 4													
Frequency	0.86		0.69, 1.06	0.154	0.86	0.70, 1.06	0.147	0.97	0.77, 1.21	0.779	0.99	0.80, 1.22	0.918
Intensity	1.20		0.92, 1.58	0.177	1.03	0.79, 1.36	0.807	1.24	0.94, 1.66	0.133	1.23	0.94, 1.65	0.146
Burden	0.84		0.64, 1.08	0.170	1.04	0.80, 1.36	0.763	0.69	0.51, 0.92	0.016	0.70	0.53, 0.91	0.010
Week 2													
Frequency	0.91		0.75, 1.09	0.305	0.95	0.78, 1.15	0.584	0.93	0.76, 1.14	0.486	0.91	0.75, 1.10	0.325
Intensity	1.20		0.94, 1.54	0.150	1.15	0.89, 1.49	0.280	1.36	1.05, 1.78	0.022	1.26	0.98, 1.62	0.076
Burden	0.76		0.60, 0.95	0.015	0.80	0.64, 1.00	0.053	0.71	0.55, 0.90	0.005	0.75	0.6, 0.94	0.014
Week 4													
Frequency	0.97		0.79, 1.18	0.746	0.85	0.69, 1.03	0.103	0.88	0.70, 1.09	0.255	0.85	0.69, 1.04	0.110
Intensity	1.05		0.81, 1.36	0.738	1.05	0.80, 1.37	0.741	1.22	0.92, 1.62	0.166	1.15	0.89, 1.51	0.290
Burden	0.81		0.64, 1.03	0.086	0.99	0.78, 1.26	0.964	0.77	0.59, 0.99	0.042	0.81	0.64, 1.03	0.085
Week 6													
Frequency	1.14		0.91, 1.43	0.251	1.01	0.82, 1.26	0.893	1.00	0.79, 1.27	0.988	1.13	0.9, 1.43	0.312
Intensity	0.68		0.50, 0.93	0.015	0.82	0.61, 1.11	0.198	1.06	0.76, 1.47	0.753	0.91	0.67, 1.24	0.543
Burden	0.85		0.63, 1.14	0.280	0.85	0.64, 1.12	0.257	0.60	0.42, 0.84	0.004	0.62	0.45, 0.83	0.002

*HRSD*_*17*_ 17-item Hamilton Rating Scale for Depression, *QIDS-SR*_*16*_ 16-item Quick Inventory of Depression Symptomatology—Self Reported, *OR* odds ratio.

### Adverse events

Four hundred and seventy-three (46.9%) participants experienced an adverse event between baseline and week 8. Supplementary Table 7 shows the distribution of adverse events related to different system organ classes. Supplementary Table 8 shows the distribution of individually reported adverse events. One thousand and sixteen adverse events were reported in iSPOT-D. Of these, 804 (79.1%) were reported as being related to the antidepressant treatments. The most common adverse events reported that were likely related to the study treatments were psychiatric disorders (28.6%, 230/804), gastrointestinal disorders (27.6%, 222/804) nervous system disorders (16.3%, 131/804), general disorders (12.1%, 97/804), and metabolism and nutrition disorders (5.7%, 46/804). However, the presence of these adverse events were not associated with HRSD_17_ or QIDS-SR_16_ remission and response (all *p* > 0.05).

## Discussion

These findings are the first to comprehensively outline the progression of side effect severity throughout the course of antidepressant treatment and their impact on treatment outcome. We found side effects were greatest at week 2, then gradually decreased up to week 6. Treatment type and anxiety symptoms did not impact the severity of side effects over the course of treatment. Finally, burden of side effects—but not frequency or intensity of side effects—were associated with poorer treatment outcome and as early as 4 days post-treatment.

Frequency, intensity, and burden of side effects increased from day 4 to week 2 post treatment. However, only frequency and intensity of side effects decreased after 4 weeks of treatment. Identifying the point at which side effects begins to recede has important clinical implications, as it may take up to 6 weeks for patients to begin seeing a reduction in side effect frequency and intensity. However, burden of side effects did not decrease after 4 weeks of treatment and endured throughout the course of antidepressant treatment. The enduring perceived burden of side effects is a commonly reported issue that contributes to the myriad of issues related to treatment adherence and efficacy [36]. Furthermore, depression severity was only weakly correlated with side effects, suggesting that the reduction of frequency and intensity of side effects was not just a proxy of a reduction in depression severity. Taken together, burden of side effects fail to decrease over the course of treatment and should be considered when assessing treatment suitability for patients in clinical practice.

Previous studies suggest that treatment type and anxiety symptoms may impact the severity of antidepressant side effects [8, 12]. However, treatment type, anxiety symptom severity, and the presence of anxious depression did not impact the severity of side effects in the current study. For example, previous studies have found a greater maximum frequency, intensity, and burden of side effects compared to people with non-anxious depression [12] and a greater side effect severity at study endpoints [13]. Here we investigated both maximum reported side effects and the change in severity of side effects at multiple study timepoints in people with anxious and non-anxious depression over the course of treatment while controlling for multiple covariates. Given that we found no differences in either the maximum reported side effects or frequency, intensity, and burden of side effects across timepoints between people with anxious and non-anxious depression, our difference in findings may be attributable to either a difference in the clinical severity of samples or lack of covariate controls. However, not all previous results are at odds with the current findings. For example, Gaspersz et al. [14]. found that syndromal anxious depression did not predict severity of side effects. Taken together, these results suggest that anxious symptom severity and the presence of anxious depression do not impact the severity of side effects over the course of antidepressant treatment.

A greater burden of side effects was associated with poorer QIDS-SR_16_ remission and response at every timepoint, except for week 4 QIDS-SR_16_ response. The association with poorer QIDS-SR_16_ treatment outcome was present as early as 4 days post treatment. Moreover, the presence of adverse events were not associated with poorer treatment outcome. These results indicate that, in addition to side effects experienced throughout treatment, even initial antidepressant side effects can contribute towards poor treatment outcome. Moreover, the immediate impact of side effect burden is especially salient given depressive symptoms do not generally begin to resolve till weeks after initial treatment [37]. Associations between burden of side effects were only found for QIDS-SR_16_ and not for the HRSD_17_, which may be related to the fact that both the QIDS-SR_16_ and the FIBSER are self-reported, while the HRSD_17_ is clinician-rated. Furthermore, while the HRSD_17_ is a multidimensional construct measuring both anxiety and depression, the QIDS-SR_16_ is a unidimensional construct measuring depression alone [38]. The QIDS-SR_16_ unilateral focus on depression may also align more closely with the FIBSER side effects related to antidepressant treatment compared to the HRSD_17_. Nevertheless, greater burden of side effects presents as a prognostic marker of poorer QIDS-SR_16_ treatment outcome and should be monitored closely.

This study has several limitations. First, our findings are limited to the antidepressants used in the current study, with other antidepressants requiring further investigation. Second, while side effect severity and general classes of side effects were assessed, specific side effects were not. Furthermore, side effects could not be matched with their corresponding severity. Therefore, the burden of some specific side effects may negatively impact treatment outcome more than others. While the FIBSER specifically targets side effects due to antidepressant medication, other protocol allowed non-psychotropic medications may have been misinterpreted for study treatment side effects and could impact the findings. Finally, because participants were diagnosed with DSM-IV criteria, the DSM-5 anxious distress specifier definition was not assessed [11].

Future research should aim to identify specific side effects that contribute to greater burden of side effect severity and poorer antidepressant treatment outcome. Given that burden of side effects not only predicted poorer treatment outcome but also failed to decrease throughout the course of treatment, a specific focus for future research should also be into the impacts of side effects causing enduring vs transient burden.

In conclusion, our results suggest that only frequency and intensity of side effects start to recede after at least 4 weeks of treatment and that anxious symptoms and the presence of anxious depression do not impact side effect severity. Furthermore, burden of side effects that are present as early as 4 days post-treatment predicts poorer treatment outcome and should be monitored closely.

## Supplementary information

Supplementary material
