# Supplementary material for: Antidepressant side effects and their impact on treatment outcome in people with major depressive disorder: an iSPOT-D report
Source: Transl Psychiatry. 2021 Aug 4;11:417. doi: 10.1038/s41398-021-01533-1 (PMC8338944; doi:10.1038/s41398-021-01533-1)
Supplement: Supplementary file 1 — Supplementary material [file 41398_2021_1533_MOESM1_ESM.docx]

Supplementary Figure 1.

*CONSORT overview of iSPOT-D MDD participants.*


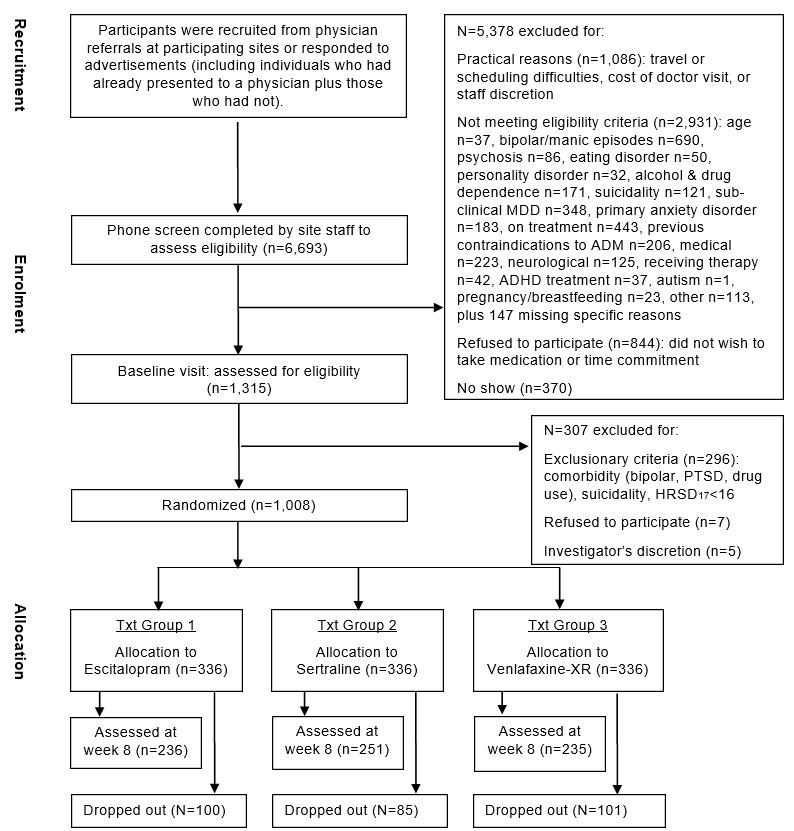


Abbreviations: ADHD, Attention-Deficit/Hyperactivity Disorder; ADM, antidepressant medication; HRSD_17_, 17-item Hamilton Rating Scale for Depression; iSPOT-D, International Study to Predict Optimized Treatment-in Depression; MDD, Major Depressive Disorder; PTSD, Posttraumatic Stress Disorder.

Supplementary Figure 2.

*iSPOT-D inclusion and exclusion criteria.*


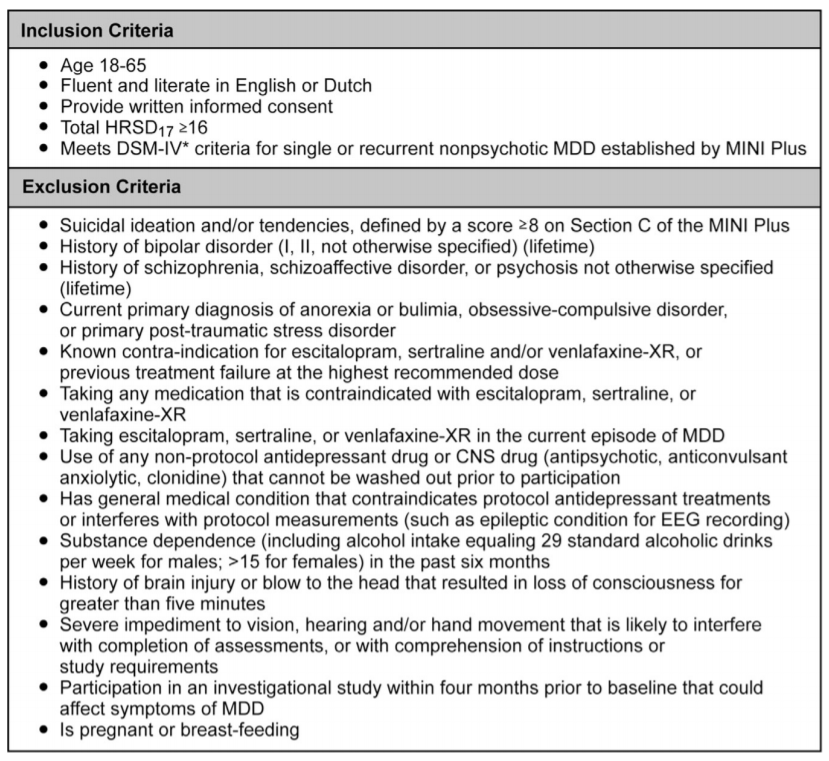


Supplementary Figure 3.

*Distribution of HRSD_17_ anxiety/somatisation factor scores.*


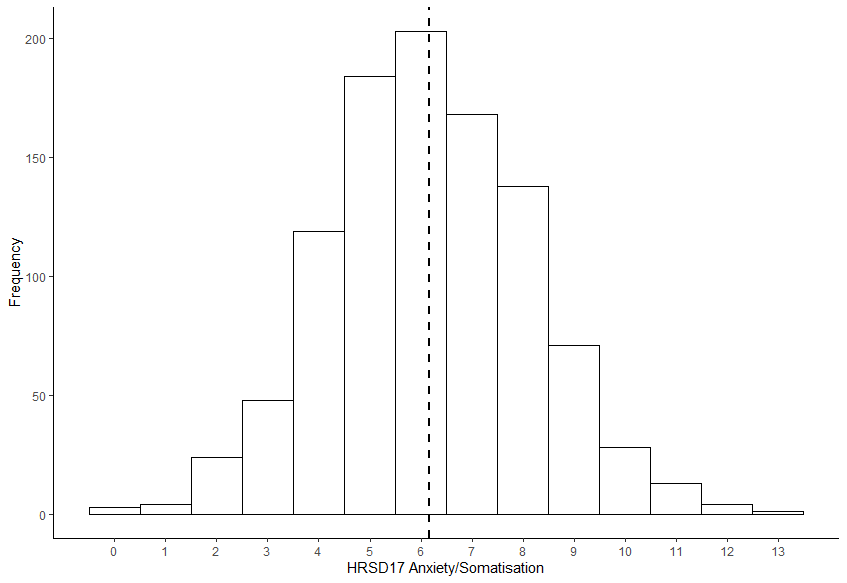


*Note: Dashed lines in histograms represent mean (x̄) HRSD_17_ anxiety/somatisation factor scores.*

Supplementary Table 1: iSPOT-D study management sites and investigators

| **Cross-site role** | |  | **Study PIs** |  |  |
| --- | --- | --- | --- | --- | --- |
| Sponsor |  |  | Evian Gordon |  |  |
| Academic |  |  | Leanne Williams | |  |
| **Type of site** | **Name of site** | **Department/Hospital or Academic Affiliation** | **Site PIs** | **Site COIs** | **Site Services** |
| **United States of America** | | | | | |
| Academic | *Stanford University, Palo Alto, CA | Psychiatry | Charles Debattista | Alan Schatzberg  Brent Solvason | Outpatient psychiatry |
|  |  |  | Amit Etkin |  |  |
| Academic | *University of St Louis, MI | Psychology | Steven Bruce | Robert Paul | Outpatient psychology |
| Academic | *Ohio State University, Columbus, OH | Psychiatry | Radu Saveanu | Anne-Marie Duchemin | Outpatient psychiatry |
| Private | *NeuroDevelopment Center, Providence, RI | Brown University | Larry Hirshberg |  | Outpatient psychology |
| Private | *ADD Treatment Center |  | Jeffrey Wilson | Garry Schummer | Outpatient psychology |
| Private | *Brain Resource Center NYC | Columbia University | Kamran Fallahpour |  | Outpatient psychology |
| Private | *Brain Resource Center NJ |  | Mona Ismail |  | Outpatient psychology |
| Private | *Shanti Clinical Trials Colton, Los Angeles, CA |  | James Rosenthal | Satish Sood  Gurmeet Multani | Outpatient psychiatry |
| Private | *Center for Healing the Human Spirit, Tarzana, Los Angeles, CA |  | Barbara Cohen |  | Outpatient psychology |
| Private | Skyland Behavioral Health Associates , NC |  | Roger deBeus |  | Psychology |
| **Australia & New Zealand** | | | | | |
| Academic | *University of Sydney | Brain Dynamics Center and General Practice, Medical School-Westmead | Anthony Harris  Tim Usherwood | Leanne Williams Phillip Boyce | Combined outpatient psychiatry and  primary care |
| Academic | *Monash University | Alfred Hospital | Jayashri Kulkarni | Paul Fitzgerald | Outpatient psychiatry |
| Academic | *Flinders University | School of Psychology | Richard Clark |  | Outpatient psychology |
| Academic | *Swinburne University | School of Psychology | Con Stough |  | Outpatient psychology |
| Academic | *University of Auckland | School of Pharmacy,  Faculty of Medical & Health Sciences | Bruce Russell | Robert Kydd | Outpatient psychiatry |
| Private | Mindmedico  Hobart TAS |  | Stuart Hooper |  | Outpatient psychiatry |
| **Netherlands** |  |  |  |  |  |
| Private | *Brainclinics Diagnostics & Treatment, Nijmegen | Nijmegen University | Martijn Arns | Niels Veth  Desiree Spronk | Outpatient psychology |
| Private | *Psy-care  Valkenburg |  | Martijn Arns | Nicole Stals | Outpatient psychology |
| **South Africa** |  |  |  |  |  |
| Private | *Brain Health, Johannesburg | University of Wittswatersrand | Elizabeth Wallis | Edward Wolff | Outpatient psychology |

Abbreviations: PI: Primary Investigator, COI: Co-Investigator

* These sites contributed data for the first 1008 MDD patients reported on in this paper

Supplementary Table 2.

*Distribution of categorical FIBSER scores at each study timepoint.*

|  | ***Frequency of the side effects due to antidepressant medication taken within the past week for depression*** | | | | | | |
| --- | --- | --- | --- | --- | --- | --- | --- |
| Frequency | ***No side***  ***effects*** | ***Present 10%***  ***of the time*** | ***Present 25%***  ***of the time*** | ***Present 50%***  ***of the time*** | ***Present 75%***  ***of the time*** | ***Present 90%***  ***of the time*** | ***Present all***  ***of the time*** |
| Time point | ***N (%)*** | ***N (%)*** | ***N (%)*** | ***N (%)*** | ***N (%)*** | ***N (%)*** | ***N (%)*** |
| Day 4 | 463 (64.8) | 94 (13.1) | 58 (8.1) | 45 (6.3) | 25 (3.5) | 15 (2.1) | 15 (2.1) |
| Week 2 | 300 (42.9) | 146 (20.9) | 99 (14.2) | 71 (10.2) | 39 (5.6) | 20 (2.9) | 24 (3.4) |
| Week 4 | 275 (40.9) | 159 (23.6) | 105 (15.6) | 60 (8.9) | 41 (6.1) | 12 (1.8) | 21 (3.1) |
| Week 6 | 286 (46.1) | 154 (24.8) | 78 (12.6) | 46 (7.4) | 26 (4.2) | 15 (2.4) | 15 (2.4) |
| Week 8 | 285 (41.3) | 198 (28.7) | 98 (14.2) | 52 (7.5) | 26 (3.8) | 16 (2.3) | 15 (2.2) |
|  | ***Intensity of the side effects due to antidepressant medication taken within the past week*** | | | | | | |
| Intensity | ***No side***  ***effects*** | ***Trivial*** | ***Mild*** | ***Moderate*** | ***Marked*** | ***Severe*** | ***Intolerable*** |
| Time point | ***N (%)*** | ***N (%)*** | ***N (%)*** | ***N (%)*** | ***N (%)*** | ***N (%)*** | ***N (%)*** |
| Day 4 | 457 (64.0) | 59 (8.3) | 103 (14.4) | 54 (7.6) | 27 (3.8) | 12 (1.7) | 2 (0.3) |
| Week 2 | 277 (39.6) | 97 (13.9) | 155 (22.2) | 116 (16.6) | 31 (4.4) | 14 (2.0) | 9 (1.3) |
| Week 4 | 258 (38.3) | 100 (14.9) | 173 (25.7) | 87 (12.9) | 42 (6.2) | 11 (1.6) | 2 (0.3) |
| Week 6 | 272 (43.9) | 97 (15.7) | 143 (23.1) | 68 (11.0) | 26 (4.2) | 13 (2.1) | 0 (0.0) |
| Week 8 | 273 (39.6) | 147 (21.3) | 141 (20.5) | 81 (11.8) | 37 (5.4) | 8 (1.2) | 2 (0.3) |
|  | ***Degree to which side effects due to antidepressant medication interfered with day-to-day functions within the past week*** | | | | | | |
| Burden | ***No***  ***Impairment*** | ***Minimal***  ***Impairment*** | ***Mild***  ***Impairment*** | ***Moderate***  ***Impairment*** | ***Marked***  ***Impairment*** | ***Severe***  ***Impairment*** | ***Unable to***  ***Function*** |
| Time point | ***N (%)*** | ***N (%)*** | ***N (%)*** | ***N (%)*** | ***N (%)*** | ***N (%)*** | ***N (%)*** |
| Day 4 | 513 (71.7) | 99 (13.8) | 43 (6) | 35 (4.9) | 15 (2.1) | 7 (1.0) | 3 (0.4) |
| Week 2 | 394 (56.4) | 127 (18.2) | 95 (13.6) | 46 (6.6) | 24 (3.4) | 13 (1.9) | 0 (0.0) |
| Week 4 | 352 (52.3) | 151 (22.4) | 92 (13.7) | 49 (7.3) | 22 (3.3) | 5 (0.7) | 2 (0.3) |
| Week 6 | 353 (56.9) | 131 (21.1) | 70 (11.3) | 38 (6.1) | 23 (3.7) | 3 (0.5) | 2 (0.3) |
| Week 8 | 383 (55.5) | 148 (21.4) | 83 (12.0) | 44 (6.4) | 25 (3.6) | 4 (0.6) | 3 (0.4) |

Supplementary Table 3.

*Distribution of last recorded FIBSER scores for participants who dropped out due to safety, tolerability, or efficacy reasons (n = 42).*

| ***Frequency*** | | |  | | ***Intensity*** | | | |  | ***Burden*** | | | |
| --- | --- | --- | --- | --- | --- | --- | --- | --- | --- | --- | --- | --- | --- |
| **Score** | ***n*** | ***%*** | |  | | **Score** | ***n*** | ***%*** |  | ***Score*** | ***n*** | ***%*** |  |
| No Side Effects | 13 | 31.0 | |  | | No Side Effects | 13 | 31.0 |  | No impairment | 12 | 28.6 |  |
| Present 10% of the time | 5 | 11.9 | |  | | Trivial side effects | 1 | 2.4 |  | Minimal Impairment | 7 | 16.7 |  |
| Present 25% of the time | 8 | 19.0 | |  | | Mild side effects | 9 | 21.4 |  | Mild impairment | 6 | 14.3 |  |
| Present 50% of the time | 5 | 11.9 | |  | | Moderate side effects | 9 | 21.4 |  | Moderate Impairment | 8 | 19.0 |  |
| Present 75% of the time | 5 | 11.9 | |  | | Marked side effects | 2 | 4.8 |  | Marked impairment | 6 | 14.3 |  |
| Present 90% of the time | 1 | 2.4 | |  | | Severe side effects | 5 | 11.9 |  | Severe Impairment | 2 | 4.8 |  |
| Present all of the time | 5 | 11.9 | |  | | Intolerable side effects | 3 | 7.1 |  | Unable to function | 1 | 2.4 |  |

Supplementary Table 4.

*Results from hierarchical mixed effect models with people who dropped out of the study due to side effects/efficacy removed comparing covariate only models to those including timepoint, and those including timepoint to those including treatment type, anxious symptom severity, and presence of anxious depression. Abbreviations: AIC, Akaike Information Criterion; BIC, Bayesian Information Criterion.*

|  |  |  | ***FIBSER - Frequency*** | | | | |  | ***FIBSER - Intensity*** | | | | | |  | ***FIBSER - Burden*** | | | | | |
| --- | --- | --- | --- | --- | --- | --- | --- | --- | --- | --- | --- | --- | --- | --- | --- | --- | --- | --- | --- | --- | --- |
| **Models** |  | ***AIC*** | | ***BIC*** | ***χ2*** | ***df*** | ***p*** | ***AIC*** | | ***BIC*** | ***χ2*** | ***df*** | ***p*** | ***AIC*** | | | ***BIC*** | ***χ2*** | ***df*** | ***P*** |  |
| Covariate Model |  | 9831.8 | | 9879.4 | - | - | - | 9297.8 | | 9345.4 | - | - | - | 8269.4 | | | 8317.1 | - | - | - |  |
| + Timepoint |  | 9777.6 | | 9849.0 | 62.18 | 4 | <.001 | 9211.9 | | 9283.3 | 93.91 | 4 | <.001 | 8220.3 | | | 8291.8 | 57.13 | 4 | <.001 |  |
| + Timepoint + Tx |  | 9778.8 | | 9862.2 | 2.74 | 2 | 0.254 | 9212.2 | | 9295.6 | 3.62 | 2 | 0.164 | 8221.2 | | | 8304.6 | 3.11 | 2 | 0.211 |  |
| + Timepoint + DASS_42_ Anxiety |  | 9779.3 | | 9856.7 | 0.29 | 1 | 0.591 | 9213.8 | | 9291.2 | 0.06 | 1 | 0.804 | 8222.0 | | | 8299.4 | 0.33 | 1 | 0.566 |  |
| + Timepoint + HRSD_17_ Anxiety/Somatization |  | 9779.2 | | 9856.6 | 0.41 | 1 | 0.520 | 9213.2 | | 9290.6 | 0.66 | 1 | 0.416 | 8219.8 | | | 8297.2 | 2.52 | 1 | 0.112 |  |
| + Timepoint + HRSD_17_ Anxious Depression |  | 9778.4 | | 9855.8 | 1.16 | 1 | 0.282 | 9213.7 | | 9291.1 | 0.17 | 1 | 0.676 | 8222.1 | | | 8299.5 | 0.21 | 1 | 0.645 |  |
| + Timepoint + Syndromal Anxious Depression |  | 9779.6 | | 9857.0 | 0.01 | 1 | 0.907 | 9213.2 | | 9290.6 | 0.64 | 1 | 0.422 | 8222.3 | | | 8299.7 | 0.04 | 1 | 0.843 |  |

Supplementary Table 5.

*Maximum reported side effects by presence of anxious depression.*

|  | **HRSD**  **Anxious Depression** | **HRSD**  **Non-Anxious Depression** |  | **Syndromal Anxious Depression** | **Syndromal Non-Anxious Depression** |  |
| --- | --- | --- | --- | --- | --- | --- |
| **Frequency** | **N (%)** | **N (%)** | ***p*** | **N (%)** | **N (%)** | ***p*** |
| No side effects | 91 (25.1) | 118 (23.8) |  | 48 (22.2) | 159 (24.8) |  |
| Present 10% of the time | 77 (21.2) | 95 (19.2) |  | 37 (17.1) | 135 (21.1) |  |
| Present 25% of the time | 57 (15.7) | 93 (18.8) |  | 34 (15.7) | 116 (18.1) |  |
| Present 50% of the time | 53 (14.6) | 76 (15.3) |  | 32 (14.8) | 97 (15.1) |  |
| Present 75% of the time | 34 (9.4) | 49 (9.9) |  | 23 (10.6) | 60 (9.4) |  |
| Present 90% of the time | 21 (5.8) | 30 (6.0) |  | 20 (9.3) | 31 (4.8) |  |
| Present all of the time | 30 (8.3) | 35 (7.1) | 0.891 | 22 (10.2) | 43 (6.7) | 0.112 |
| **Intensity** | **N (%)** | **N (%)** | ***p*** | **N (%)** | **N (%)** | ***p*** |
| No side effects | 85 (23.4) | 108 (21.8) |  | 42 (19.4) | 149 (23.2) |  |
| Trivial | 46 (12.7) | 60 (12.1) |  | 24 (11.1) | 82 (12.8) |  |
| Mild | 74 (20.4) | 127 (25.6) |  | 49 (22.7) | 152 (23.7) |  |
| Moderate | 81 (22.3) | 119 (24) |  | 56 (25.9) | 144 (22.5) |  |
| Marked | 42 (11.6) | 57 (11.5) |  | 26 (12.0) | 73 (11.4) |  |
| Severe | 26 (7.2) | 20 (4.0) |  | 13 (6.0) | 33 (5.1) |  |
| Intolerable | 9 (2.5) | 5 (1.0) | 0.148 | 6 (2.8) | 8 (1.2) | 0.562 |
| **Burden** | **N (%)** | **N (%)** | ***p*** | **N (%)** | **N (%)** | ***p*** |
| No Impairment | 127 (35) | 163 (32.9) |  | 63 (29.2) | 225 (35.1) |  |
| Minimal Impairment | 67 (18.5) | 123 (24.8) |  | 45 (20.8) | 145 (22.6) |  |
| Mild Impairment | 64 (17.6) | 95 (19.2) |  | 38 (17.6) | 121 (18.9) |  |
| Moderate Impairment | 52 (14.3) | 69 (13.9) |  | 40 (18.5) | 81 (12.6) |  |
| Marked Impairment | 36 (9.9) | 31 (6.2) |  | 20 (9.3) | 47 (7.3) |  |
| Severe Impairment | 11 (3.0) | 11 (2.2) |  | 7 (3.2) | 15 (2.3) |  |
| Unable to Function | 6 (1.7) | 4 (0.8) | 0.135 | 3 (1.4) | 7 (1.1) | 0.264 |

Supplementary Table 6.

*Post-hoc test results.*

|  |  | ***FIBSER - Frequency*** | | | ***FIBSER - Intensity*** | | | ***FIBSER - Burden*** | | | |
| --- | --- | --- | --- | --- | --- | --- | --- | --- | --- | --- | --- |
| **Comparison** |  | ***t*** | ***p*** | ***p**** | ***t*** | ***p*** | ***p**** | ***t*** | ***p*** | ***p**** |  |
| Day 4 – Week 2 |  | -7.13 | <.001 | <.001 | -8.66 | <.001 | <.001 | -6.54 | <.001 | <.001 |  |
| Day 4 – Week 4 |  | -6.47 | <.001 | <.001 | -8.33 | <.001 | <.001 | -6.50 | <.001 | <.001 |  |
| Day 4 – Week 6 |  | -3.73 | <.001 | <.001 | -5.29 | <.001 | <.001 | -4.98 | <.001 | <.001 |  |
| Day 4 – Week 8 |  | -4.61 | <.001 | <.001 | -6.12 | <.001 | <.001 | -5.72 | <.001 | <.001 |  |
| Week 2 – Week 4 |  | 0.66 | 0.507 | 0.507 | 0.33 | 0.744 | 0.744 | 0.02 | 0.980 | 0.980 |  |
| Week 2 – Week 6 |  | 3.30 | 0.001 | 0.002 | 3.24 | 0.001 | 0.002 | 1.44 | 0.151 | 0.263 |  |
| Week 2 – Week 8 |  | 2.70 | 0.007 | 0.012 | 2.75 | 0.006 | 0.009 | 0.96 | 0.338 | 0.439 |  |
| Week 4 – Week 6 |  | 2.65 | 0.008 | 0.012 | 2.92 | 0.004 | 0.006 | 1.41 | 0.158 | 0.263 |  |
| Week 4 – Week 8 |  | 2.01 | 0.044 | 0.055 | 2.41 | 0.016 | 0.020 | 0.93 | 0.351 | 0.439 |  |
| Week 6 – Week 8 |  | -0.73 | 0.464 | 0.507 | -0.61 | 0.539 | 0.599 | -0.53 | 0.597 | 0.663 |  |

** C*orrected for using the Bonferroni-Holm (1979) method.

Supplementary Table 7.

*Distribution of adverse events by system organ classes.*

|  | ***Total*** | ***Related to***  ***Study Treatment*** |
| --- | --- | --- |
| **System Organ Classes** | ***N (%)*** | ***N (%)*** |
| Cardiac disorders | 10 (1) | 9 (1.1) |
| Endocrine disorders | 1 (0.1) | 0 (0) |
| Eye disorders | 5 (0.5) | 3 (0.4) |
| Gastrointestinal disorders | 255 (25.1) | 222 (27.6) |
| General disorders and administration site conditions | 109 (10.7) | 97 (12.1) |
| Immune system disorders | 9 (0.9) | 0 (0) |
| Infections and infestations | 48 (4.7) | 2 (0.2) |
| Injury, poisoning and procedural complications | 9 (0.9) | 0 (0) |
| Investigations | 15 (1.5) | 7 (0.9) |
| Metabolism and nutrition disorders | 48 (4.7) | 46 (5.7) |
| Musculoskeletal and connective tissue disorders | 20 (2) | 8 (1) |
| Neoplasms benign, malignant and unspecified | 1 (0.1) | 0 (0) |
| Nervous system disorders | 159 (15.6) | 131 (16.3) |
| Pregnancy, puerperium and perinatal conditions | 2 (0.2) | 0 (0) |
| Psychiatric disorders | 255 (25.1) | 230 (28.6) |
| Renal and urinary disorders | 5 (0.5) | 5 (0.6) |
| Reproductive system and breast disorders | 5 (0.5) | 4 (0.5) |
| Respiratory, thoracic and mediastinal disorders | 13 (1.3) | 5 (0.6) |
| Skin and subcutaneous tissue disorders | 4 (0.4) | 1 (0.1) |
| Vascular disorders | 43 (4.2) | 34 (4.2) |

Supplementary Table 8.

*Distribution of individually reported adverse events.*

|  | **Total** | **Related to**  **Study Treatment** |
| --- | --- | --- |
| **Adverse Event** | **N (%)** | **N (%)** |
| empty reaching | 1 (0.1) | 1 (0.1) |
| acid reflux | 2 (0.2) | 1 (0.1) |
| ADHD | 1 (0.1) | 0 (0) |
| Agitation | 2 (0.2) | 2 (0.2) |
| allergies | 2 (0.2) | 0 (0) |
| Allergies | 1 (0.1) | 0 (0) |
| allergy | 1 (0.1) | 0 (0) |
| Allergy | 1 (0.1) | 0 (0) |
| allergy breathing problems | 1 (0.1) | 0 (0) |
| Allergy Symptoms | 1 (0.1) | 0 (0) |
| Angi0 (0) | 1 (0.1) | 1 (0.1) |
| anxiety | 18 (1.8) | 17 (2.1) |
| Anxiety | 6 (0.6) | 4 (0.5) |
| Anxiety increased | 1 (0.1) | 0 (0) |
| Anxious/agitated | 1 (0.1) | 1 (0.1) |
| anxiousness | 1 (0.1) | 1 (0.1) |
| APHTHOUS ULCERS | 1 (0.1) | 0 (0) |
| appetite decrease | 3 (0.3) | 3 (0.4) |
| appetite decreased | 2 (0.2) | 2 (0.2) |
| Appetite decreased | 1 (0.1) | 1 (0.1) |
| appetite increase | 1 (0.1) | 1 (0.1) |
| Appetite increase | 1 (0.1) | 1 (0.1) |
| appetite lost | 1 (0.1) | 1 (0.1) |
| arm infection | 1 (0.1) | 0 (0) |
| Attempted Suicide | 1 (0.1) | 1 (0.1) |
| Axiety | 1 (0.1) | 1 (0.1) |
| Back pain | 1 (0.1) | 0 (0) |
| Backpain worse | 1 (0.1) | 0 (0) |
| bloating | 1 (0.1) | 1 (0.1) |
| blurry eyes | 1 (0.1) | 1 (0.1) |
| blurry vision | 1 (0.1) | 1 (0.1) |
| Blurry vision | 1 (0.1) | 1 (0.1) |
| bodily discomfort | 1 (0.1) | 1 (0.1) |
| body aches | 1 (0.1) | 0 (0) |
| Breathlessness | 1 (0.1) | 1 (0.1) |
| Broken ankle | 1 (0.1) | 0 (0) |
| Broken toe, infection | 1 (0.1) | 0 (0) |
| Bronchial asthma | 1 (0.1) | 0 (0) |
| Bronchitis | 1 (0.1) | 0 (0) |
| bruised knee | 1 (0.1) | 0 (0) |
| Burning in digestive tract | 1 (0.1) | 1 (0.1) |
| burns | 1 (0.1) | 0 (0) |
| Bursts of energy | 1 (0.1) | 1 (0.1) |
| car accident | 1 (0.1) | 0 (0) |
| Carcinoma | 1 (0.1) | 0 (0) |
| Carpal Tunnel Surgery | 1 (0.1) | 0 (0) |
| Chest infection | 1 (0.1) | 0 (0) |
| chest pain | 1 (0.1) | 1 (0.1) |
| Chest pain | 1 (0.1) | 1 (0.1) |
| Clenching Jaw | 1 (0.1) | 1 (0.1) |
| cluster headackes | 1 (0.1) | 0 (0) |
| cold | 4 (0.4) | 0 (0) |
| Cold | 8 (0.8) | 0 (0) |
| Cold symptoms | 1 (0.1) | 1 (0.1) |
| Cold/influenza | 1 (0.1) | 0 (0) |
| Cold/Influenza | 1 (0.1) | 0 (0) |
| concussion due to head injury, loss of consciousness | 1 (0.1) | 0 (0) |
| constipation | 8 (0.8) | 8 (1) |
| Constipation | 2 (0.2) | 2 (0.2) |
| Cracked Tooth | 1 (0.1) | 0 (0) |
| Crying | 1 (0.1) | 1 (0.1) |
| Decrease Appetite | 1 (0.1) | 1 (0.1) |
| Decrease in Libido | 1 (0.1) | 1 (0.1) |
| decreased appetite | 2 (0.2) | 2 (0.2) |
| Decreased appetite | 2 (0.2) | 2 (0.2) |
| Decreased Appetite | 2 (0.2) | 2 (0.2) |
| decreased concentration | 1 (0.1) | 1 (0.1) |
| Decreased libido | 2 (0.2) | 2 (0.2) |
| Decreased Libido | 2 (0.2) | 1 (0.1) |
| Decreased sex drive | 1 (0.1) | 1 (0.1) |
| decreased sexual feelings | 1 (0.1) | 1 (0.1) |
| decreased sleep | 1 (0.1) | 1 (0.1) |
| Diaherra | 2 (0.2) | 1 (0.1) |
| DIARRHEA - INTERMITTENT | 1 (0.1) | 0 (0) |
| diarrhea | 9 (0.9) | 9 (1.1) |
| Diarrhea | 8 (0.8) | 7 (0.9) |
| diarrhia | 1 (0.1) | 1 (0.1) |
| diarrhoea | 3 (0.3) | 3 (0.4) |
| Diarrhoea | 2 (0.2) | 2 (0.2) |
| diarrohea | 1 (0.1) | 1 (0.1) |
| Diarrohea/ stomach cramping | 1 (0.1) | 1 (0.1) |
| dicreased appetite | 1 (0.1) | 1 (0.1) |
| Difficult to orgasm | 1 (0.1) | 1 (0.1) |
| Difficulty breathing | 1 (0.1) | 1 (0.1) |
| Difficulty falling asleep | 1 (0.1) | 1 (0.1) |
| Difficulty getting to sleep (INSOMNIA) | 1 (0.1) | 1 (0.1) |
| difficulty sleeping | 1 (0.1) | 0 (0) |
| dilated eyes | 1 (0.1) | 1 (0.1) |
| Dilated pupils | 1 (0.1) | 1 (0.1) |
| Disorientation | 1 (0.1) | 1 (0.1) |
| Disrupted sleep | 8 (0.8) | 8 (1) |
| diziness | 1 (0.1) | 1 (0.1) |
| Diziness | 2 (0.2) | 2 (0.2) |
| dizziness | 8 (0.8) | 7 (0.9) |
| Dizziness | 9 (0.9) | 8 (1) |
| Dizzy | 3 (0.3) | 3 (0.4) |
| Dizzy (occasio0 (0)l) | 1 (0.1) | 1 (0.1) |
| Dizzy (occasio0 (0)lly) | 1 (0.1) | 1 (0.1) |
| Dizzy on wakening | 1 (0.1) | 1 (0.1) |
| dizzyness | 4 (0.4) | 3 (0.4) |
| Dizzyness | 2 (0.2) | 2 (0.2) |
| Drops in energy mid-day | 1 (0.1) | 1 (0.1) |
| drowsiness | 9 (0.9) | 9 (1.1) |
| Drowsiness | 6 (0.6) | 5 (0.6) |
| drowsy | 1 (0.1) | 1 (0.1) |
| Drowsy | 1 (0.1) | 1 (0.1) |
| drowsy, mentally foggy | 1 (0.1) | 1 (0.1) |
| dry mouth | 1 (0.1) | 1 (0.1) |
| Dry Lips | 1 (0.1) | 1 (0.1) |
| dry mouth | 20 (2) | 20 (2.5) |
| Dry mouth | 12 (1.2) | 12 (1.5) |
| Dry Mouth | 4 (0.4) | 3 (0.4) |
| DRY MOUTH | 1 (0.1) | 1 (0.1) |
| Ear infection | 1 (0.1) | 0 (0) |
| edginess | 1 (0.1) | 1 (0.1) |
| Elevated mood | 1 (0.1) | 1 (0.1) |
| Endoscopy | 1 (0.1) | 0 (0) |
| erectile dysfunction | 1 (0.1) | 1 (0.1) |
| Erectile dysfunction | 1 (0.1) | 1 (0.1) |
| Erectile/sexual dysfunction | 1 (0.1) | 1 (0.1) |
| Excess sweating | 1 (0.1) | 1 (0.1) |
| excessive Perspiration | 1 (0.1) | 1 (0.1) |
| Excessive sleep | 1 (0.1) | 1 (0.1) |
| Excessive sweating at night | 1 (0.1) | 1 (0.1) |
| excessively tired | 1 (0.1) | 1 (0.1) |
| Exhaustion | 1 (0.1) | 1 (0.1) |
| experiences more emotions | 1 (0.1) | 1 (0.1) |
| Eye infection | 1 (0.1) | 0 (0) |
| Eye strain | 1 (0.1) | 0 (0) |
| fainted | 1 (0.1) | 1 (0.1) |
| faintness | 1 (0.1) | 1 (0.1) |
| fast heart beat | 1 (0.1) | 1 (0.1) |
| Fast pulse readings | 1 (0.1) | 0 (0) |
| fatigue | 10 (1) | 10 (1.2) |
| Fatigue | 14 (1.4) | 13 (1.6) |
| feel hyper | 1 (0.1) | 1 (0.1) |
| feeling drowsy | 1 (0.1) | 1 (0.1) |
| Feeling lightheaded | 1 (0.1) | 1 (0.1) |
| Feeling more negative/demotivated | 1 (0.1) | 1 (0.1) |
| feeling of less body control | 1 (0.1) | 1 (0.1) |
| Feeling slow | 1 (0.1) | 1 (0.1) |
| feeling tired | 2 (0.2) | 2 (0.2) |
| Feeling tired. | 1 (0.1) | 1 (0.1) |
| Feeling uncomfortable in new residence | 1 (0.1) | 0 (0) |
| feelings of feeling trapped inside her body, locked up | 1 (0.1) | 0 (0) |
| Feelings of Paranoia | 1 (0.1) | 1 (0.1) |
| feelling much better | 1 (0.1) | 1 (0.1) |
| Feels tired | 1 (0.1) | 1 (0.1) |
| felt tired | 1 (0.1) | 1 (0.1) |
| Fever | 1 (0.1) | 0 (0) |
| fever intermitant | 1 (0.1) | 0 (0) |
| Flu | 2 (0.2) | 0 (0) |
| Flu symptoms | 1 (0.1) | 0 (0) |
| Fluctuating memory | 1 (0.1) | 1 (0.1) |
| Fluid build up in legs | 1 (0.1) | 1 (0.1) |
| Food Poisoning. | 1 (0.1) | 0 (0) |
| Food Posioning | 1 (0.1) | 0 (0) |
| Forgetful | 2 (0.2) | 2 (0.2) |
| Frequent, soft stools | 1 (0.1) | 1 (0.1) |
| Funny taste in mouth | 1 (0.1) | 1 (0.1) |
| Fuzzy head | 1 (0.1) | 1 (0.1) |
| gas | 1 (0.1) | 1 (0.1) |
| gastrointersti0 (0)l problems | 1 (0.1) | 1 (0.1) |
| gastrointesti0 (0)l upset | 1 (0.1) | 1 (0.1) |
| Gastroscopy | 1 (0.1) | 0 (0) |
| Giddy/dizzy | 1 (0.1) | 1 (0.1) |
| Gland pain | 1 (0.1) | 0 (0) |
| Grief - family problems, deaths, cancer, etc. | 1 (0.1) | 0 (0) |
| Grinding teeth | 1 (0.1) | 1 (0.1) |
| Gum Infection | 1 (0.1) | 0 (0) |
| gum pain | 1 (0.1) | 0 (0) |
| Hand shaking | 1 (0.1) | 1 (0.1) |
| Harder to focus | 1 (0.1) | 0 (0) |
| hay fever | 1 (0.1) | 0 (0) |
| Hazy feeling | 1 (0.1) | 1 (0.1) |
| Head cold | 2 (0.2) | 0 (0) |
| head injury | 1 (0.1) | 0 (0) |
| headache-tightness | 1 (0.1) | 1 (0.1) |
| headache | 30 (3) | 26 (3.2) |
| Headache | 26 (2.6) | 18 (2.2) |
| HEADACHE | 1 (0.1) | 0 (0) |
| headache (occasio0 (0)l) | 1 (0.1) | 1 (0.1) |
| headache at back of head | 1 (0.1) | 1 (0.1) |
| headaches | 3 (0.3) | 3 (0.4) |
| Headaches | 20 (2) | 17 (2.1) |
| Headaches (Migranes) | 1 (0.1) | 0 (0) |
| Headaches (occasio0 (0)l) | 2 (0.2) | 2 (0.2) |
| Headaches/migraines | 1 (0.1) | 1 (0.1) |
| headacke | 1 (0.1) | 1 (0.1) |
| Headacke | 1 (0.1) | 1 (0.1) |
| heart palpitations | 1 (0.1) | 1 (0.1) |
| Heart palpitations | 1 (0.1) | 1 (0.1) |
| Heart Palpitations | 1 (0.1) | 1 (0.1) |
| Heaviness in limbs | 1 (0.1) | 1 (0.1) |
| heavy sweating / cold sweat | 1 (0.1) | 1 (0.1) |
| high blood pressure | 1 (0.1) | 0 (0) |
| High Blood Pressure | 1 (0.1) | 1 (0.1) |
| high cholesterol | 1 (0.1) | 0 (0) |
| High holesterol level | 1 (0.1) | 0 (0) |
| higher heart rate | 1 (0.1) | 1 (0.1) |
| Hip aches | 1 (0.1) | 0 (0) |
| Hostility | 1 (0.1) | 1 (0.1) |
| Hot flashes | 1 (0.1) | 1 (0.1) |
| Hyperactive | 1 (0.1) | 1 (0.1) |
| hyperactivity | 2 (0.2) | 2 (0.2) |
| hyperhydrosis | 1 (0.1) | 1 (0.1) |
| hypersomnia | 2 (0.2) | 2 (0.2) |
| Hypersomnia | 1 (0.1) | 1 (0.1) |
| Hypersomnia/fatigue | 1 (0.1) | 1 (0.1) |
| Hypertension | 2 (0.2) | 1 (0.1) |
| in the morning extreme fatigue | 1 (0.1) | 1 (0.1) |
| I0 (0)bility to orgasm | 1 (0.1) | 1 (0.1) |
| I0 (0)bility to orgasm a couple of times | 1 (0.1) | 1 (0.1) |
| I0 (0)bility to sleep from stress of moving away from crazy roommate | 1 (0.1) | 0 (0) |
| Increase apetite | 3 (0.3) | 3 (0.4) |
| Increase Appetite | 1 (0.1) | 1 (0.1) |
| Increased agoraphobia | 1 (0.1) | 1 (0.1) |
| Increased anxiety | 1 (0.1) | 1 (0.1) |
| Increased apetite | 1 (0.1) | 1 (0.1) |
| increased appetite | 3 (0.3) | 3 (0.4) |
| Increased appetite | 1 (0.1) | 0 (0) |
| Increased depression and grieving - very close to grandmother who died | 1 (0.1) | 0 (0) |
| Increased depression; sadness | 1 (0.1) | 0 (0) |
| increased energy | 1 (0.1) | 1 (0.1) |
| increased energy level | 1 (0.1) | 1 (0.1) |
| Increased heart rate | 1 (0.1) | 1 (0.1) |
| increased need for sleep | 1 (0.1) | 1 (0.1) |
| increased tiredness | 1 (0.1) | 1 (0.1) |
| Increased weight | 1 (0.1) | 1 (0.1) |
| indigestion | 1 (0.1) | 1 (0.1) |
| Indigestion | 1 (0.1) | 1 (0.1) |
| Influenza | 10 (1) | 0 (0) |
| Injured thumb | 1 (0.1) | 0 (0) |
| insensitive emotion | 1 (0.1) | 1 (0.1) |
| insomia | 1 (0.1) | 1 (0.1) |
| insomnia | 13 (1.3) | 8 (1) |
| Insomnia | 28 (2.8) | 26 (3.2) |
| INSOMNIA | 9 (0.9) | 5 (0.6) |
| insomnia middle | 1 (0.1) | 1 (0.1) |
| Intesti0 (0)l blockage | 1 (0.1) | 0 (0) |
| Irritability | 4 (0.4) | 4 (0.5) |
| Irritable bowel syndrome | 1 (0.1) | 0 (0) |
| Itching in hands and legs | 1 (0.1) | 0 (0) |
| Jaw clenching | 2 (0.2) | 2 (0.2) |
| Jaw stiffness | 1 (0.1) | 1 (0.1) |
| Jittery | 1 (0.1) | 1 (0.1) |
| Jittery jaw | 1 (0.1) | 1 (0.1) |
| Joint Pain/Sorness | 1 (0.1) | 0 (0) |
| Kidney infection | 1 (0.1) | 0 (0) |
| Knee pain from new cycling shoes; subject suspects plica syndrome | 1 (0.1) | 0 (0) |
| lack of concentration | 2 (0.2) | 2 (0.2) |
| lack of concetration | 1 (0.1) | 1 (0.1) |
| lack of energy | 1 (0.1) | 1 (0.1) |
| Lack of sleep | 1 (0.1) | 1 (0.1) |
| Large arachnoid cyst in left middle cranial fossa | 1 (0.1) | 0 (0) |
| lead head feeling | 1 (0.1) | 1 (0.1) |
| leg infection | 1 (0.1) | 0 (0) |
| Leg shaking | 1 (0.1) | 1 (0.1) |
| Less focused and more distracted. | 1 (0.1) | 1 (0.1) |
| less reaction abilities | 1 (0.1) | 1 (0.1) |
| less sleep | 1 (0.1) | 1 (0.1) |
| less sleeping | 1 (0.1) | 1 (0.1) |
| Lethargic | 1 (0.1) | 1 (0.1) |
| Lethargy | 2 (0.2) | 2 (0.2) |
| Lethargy/feeling slowed down, especially in the morning. | 1 (0.1) | 1 (0.1) |
| Lethargy/tiredness | 1 (0.1) | 1 (0.1) |
| Libido Decrease | 1 (0.1) | 1 (0.1) |
| LIGHTHEADED | 1 (0.1) | 0 (0) |
| lightheadedness | 1 (0.1) | 0 (0) |
| Lightheadedness | 3 (0.3) | 2 (0.2) |
| loose bowels | 1 (0.1) | 1 (0.1) |
| loss of appetite | 4 (0.4) | 4 (0.5) |
| Loss of appetite | 11 (1.1) | 11 (1.4) |
| Loss of Appetite | 2 (0.2) | 2 (0.2) |
| Loss of libido | 1 (0.1) | 1 (0.1) |
| lost weight | 1 (0.1) | 1 (0.1) |
| Low blood preassure | 1 (0.1) | 0 (0) |
| low energy | 2 (0.2) | 2 (0.2) |
| Low energy | 2 (0.2) | 2 (0.2) |
| low energy level | 1 (0.1) | 1 (0.1) |
| low libido | 2 (0.2) | 2 (0.2) |
| Low vitamin B measurement | 1 (0.1) | 0 (0) |
| lump in throat | 1 (0.1) | 1 (0.1) |
| Memory problems | 1 (0.1) | 1 (0.1) |
| Menstrual Cramps | 1 (0.1) | 0 (0) |
| Menstruation Disturbances | 1 (0.1) | 0 (0) |
| mental confusion | 1 (0.1) | 1 (0.1) |
| mental slowdown | 1 (0.1) | 1 (0.1) |
| Middle insomnia | 1 (0.1) | 1 (0.1) |
| Middle Insomnia | 1 (0.1) | 0 (0) |
| migraine | 1 (0.1) | 0 (0) |
| migraine, extreme headache | 1 (0.1) | 0 (0) |
| Mild constipation | 1 (0.1) | 1 (0.1) |
| mild dizzyness | 1 (0.1) | 1 (0.1) |
| Mild fatigue | 1 (0.1) | 1 (0.1) |
| Mild 0 (0)usea | 1 (0.1) | 1 (0.1) |
| Miscarriage | 1 (0.1) | 0 (0) |
| Mood swings | 1 (0.1) | 1 (0.1) |
| More binge eating | 1 (0.1) | 1 (0.1) |
| More difficulty sleeping | 1 (0.1) | 1 (0.1) |
| more energy / restless feeling | 1 (0.1) | 1 (0.1) |
| more sleeping | 1 (0.1) | 1 (0.1) |
| more tired | 1 (0.1) | 1 (0.1) |
| More tiredness | 1 (0.1) | 0 (0) |
| Morning anxiety | 1 (0.1) | 1 (0.1) |
| Morning headaches | 1 (0.1) | 1 (0.1) |
| morning insomnia | 1 (0.1) | 1 (0.1) |
| mouth infection | 1 (0.1) | 0 (0) |
| Muscle twitching | 1 (0.1) | 1 (0.1) |
| 0 (0)esea | 1 (0.1) | 1 (0.1) |
| 0 (0)usea | 49 (4.8) | 46 (5.7) |
| 0 (0)usea | 63 (6.2) | 58 (7.2) |
| 0 (0)usea (in the morning) | 1 (0.1) | 1 (0.1) |
| 0 (0)usea/vomiting | 1 (0.1) | 1 (0.1) |
| 0 (0)ussea | 1 (0.1) | 1 (0.1) |
| Neck pain - lump in throat | 1 (0.1) | 1 (0.1) |
| neck pain | 1 (0.1) | 0 (0) |
| Neck pain | 1 (0.1) | 0 (0) |
| negative thoughts | 1 (0.1) | 1 (0.1) |
| nervosity | 1 (0.1) | 1 (0.1) |
| Night sweat | 1 (0.1) | 1 (0.1) |
| Night sweats | 1 (0.1) | 1 (0.1) |
| Night Sweats | 1 (0.1) | 1 (0.1) |
| Nightmare, poor sleep | 1 (0.1) | 1 (0.1) |
| Nightmares | 1 (0.1) | 1 (0.1) |
| No appetite | 1 (0.1) | 1 (0.1) |
| nousea | 1 (0.1) | 1 (0.1) |
| Numbness (hands) | 1 (0.1) | 1 (0.1) |
| oesophageal spasm requiring hospitalisation | 1 (0.1) | 0 (0) |
| OVERACTIVE BLADDER | 1 (0.1) | 1 (0.1) |
| pain | 1 (0.1) | 0 (0) |
| Pain from dental work | 1 (0.1) | 0 (0) |
| Pain from hernia surgery | 1 (0.1) | 0 (0) |
| Pain from pulled tooth | 1 (0.1) | 0 (0) |
| palpitation | 2 (0.2) | 2 (0.2) |
| palsy (loss of sensation) | 1 (0.1) | 1 (0.1) |
| Panic attack | 1 (0.1) | 1 (0.1) |
| Panic Attack | 1 (0.1) | 0 (0) |
| Paraesthesia | 1 (0.1) | 0 (0) |
| Paranoia | 1 (0.1) | 1 (0.1) |
| Parasthesia | 2 (0.2) | 2 (0.2) |
| Participant reported signifcant fatigue which started 4-5 days after starting the medication. | 1 (0.1) | 1 (0.1) |
| passed out | 1 (0.1) | 1 (0.1) |
| persistant sadness | 1 (0.1) | 1 (0.1) |
| Pink Eye | 1 (0.1) | 0 (0) |
| pneumonia | 1 (0.1) | 0 (0) |
| polyuria | 1 (0.1) | 1 (0.1) |
| Polyuria | 1 (0.1) | 1 (0.1) |
| poor concentration | 1 (0.1) | 1 (0.1) |
| Poor concentration | 1 (0.1) | 1 (0.1) |
| Poor Proprioception | 1 (0.1) | 1 (0.1) |
| Poor Sleep | 1 (0.1) | 1 (0.1) |
| poor sleep again - mild | 1 (0.1) | 1 (0.1) |
| poor sleep onset | 1 (0.1) | 1 (0.1) |
| Preg0 (0)ncy | 1 (0.1) | 0 (0) |
| pressure in head worsening | 1 (0.1) | 0 (0) |
| Pressure on breast | 1 (0.1) | 1 (0.1) |
| Problems with erection | 2 (0.2) | 2 (0.2) |
| Racing Heart rate | 1 (0.1) | 1 (0.1) |
| Rash | 1 (0.1) | 1 (0.1) |
| Rash (hands after gardening) | 1 (0.1) | 0 (0) |
| RASH ON FACE | 1 (0.1) | 0 (0) |
| Red marks on scalp | 1 (0.1) | 0 (0) |
| reduced appetite | 1 (0.1) | 1 (0.1) |
| reduced effect of alcohol | 1 (0.1) | 1 (0.1) |
| Reflux | 1 (0.1) | 1 (0.1) |
| restlesness | 1 (0.1) | 1 (0.1) |
| restless feeling | 1 (0.1) | 1 (0.1) |
| Restless leg | 1 (0.1) | 1 (0.1) |
| Restless legs | 1 (0.1) | 1 (0.1) |
| Restless sleep | 3 (0.3) | 3 (0.4) |
| Restless Sleep | 1 (0.1) | 1 (0.1) |
| restless thoughts | 1 (0.1) | 1 (0.1) |
| Restlessness | 2 (0.2) | 2 (0.2) |
| runny nose | 1 (0.1) | 0 (0) |
| Seaso0 (0)l Allergies | 1 (0.1) | 0 (0) |
| Sedated from hydrogyzin - reduced dose to half | 1 (0.1) | 0 (0) |
| Severe worsening of depressive symptoms | 1 (0.1) | 1 (0.1) |
| sexual disfunction | 18 (1.8) | 18 (2.2) |
| Sexual disfunction | 2 (0.2) | 2 (0.2) |
| sexual distrbances | 1 (0.1) | 1 (0.1) |
| sexual dysfunction | 2 (0.2) | 2 (0.2) |
| Sexual Side Effects | 1 (0.1) | 1 (0.1) |
| shakiness | 2 (0.2) | 2 (0.2) |
| shaking | 1 (0.1) | 1 (0.1) |
| shaking hands | 1 (0.1) | 1 (0.1) |
| Shaky hands | 1 (0.1) | 1 (0.1) |
| shortness of breath | 1 (0.1) | 1 (0.1) |
| Sighing | 1 (0.1) | 1 (0.1) |
| Sinus Congestion | 1 (0.1) | 0 (0) |
| Sinus Headache | 1 (0.1) | 0 (0) |
| Sinus Infection | 1 (0.1) | 0 (0) |
| Sleep - early morning waking | 1 (0.1) | 1 (0.1) |
| Sleep - waking during the night | 1 (0.1) | 1 (0.1) |
| sleep distrbance | 1 (0.1) | 1 (0.1) |
| sleep distrbances | 1 (0.1) | 1 (0.1) |
| sleep distrurbances | 1 (0.1) | 1 (0.1) |
| Sleep disturbance - middle night awakening | 1 (0.1) | 1 (0.1) |
| sleep disturbance | 8 (0.8) | 8 (1) |
| Sleep disturbance | 2 (0.2) | 2 (0.2) |
| Sleep Disturbance | 1 (0.1) | 1 (0.1) |
| sleep disturbances | 1 (0.1) | 1 (0.1) |
| Sleep disturbed in middle of night - awake an hour and then back to sleep | 1 (0.1) | 1 (0.1) |
| sleep faster | 1 (0.1) | 1 (0.1) |
| Sleep worse | 1 (0.1) | 1 (0.1) |
| Sleepier / 0 (0)pping more | 1 (0.1) | 1 (0.1) |
| sleepiness | 1 (0.1) | 1 (0.1) |
| Sleepiness | 2 (0.2) | 2 (0.2) |
| sleeping a lot more | 1 (0.1) | 1 (0.1) |
| sleeping disturbance | 1 (0.1) | 1 (0.1) |
| sleeping problems | 2 (0.2) | 2 (0.2) |
| Sleeping problems | 1 (0.1) | 1 (0.1) |
| sleeplessness | 2 (0.2) | 2 (0.2) |
| Sleeplessness | 2 (0.2) | 2 (0.2) |
| Sleepy | 2 (0.2) | 2 (0.2) |
| Sleepy/tired | 1 (0.1) | 1 (0.1) |
| Slight Anxiety | 1 (0.1) | 1 (0.1) |
| Slight dry mouth | 1 (0.1) | 1 (0.1) |
| slightly headacke | 1 (0.1) | 1 (0.1) |
| slightly 0 (0)usea | 1 (0.1) | 1 (0.1) |
| slow speech | 1 (0.1) | 1 (0.1) |
| Slower mentally | 1 (0.1) | 1 (0.1) |
| Somnolence | 1 (0.1) | 1 (0.1) |
| Sore Throat | 1 (0.1) | 0 (0) |
| SORE THROAT | 1 (0.1) | 0 (0) |
| Spacey/out of body feeling | 1 (0.1) | 1 (0.1) |
| Stiff jaw | 2 (0.2) | 2 (0.2) |
| stomach ache | 4 (0.4) | 3 (0.4) |
| Stomach Ache | 1 (0.1) | 1 (0.1) |
| STOMACH ACHE | 1 (0.1) | 1 (0.1) |
| stomach cramps | 2 (0.2) | 2 (0.2) |
| Stomach cramps | 1 (0.1) | 1 (0.1) |
| stomach flu | 1 (0.1) | 0 (0) |
| stomach infection | 1 (0.1) | 0 (0) |
| Stomach infection | 1 (0.1) | 0 (0) |
| stomach pain | 2 (0.2) | 2 (0.2) |
| stomach pains | 1 (0.1) | 1 (0.1) |
| Stomach Pains | 1 (0.1) | 1 (0.1) |
| stomach upset | 2 (0.2) | 1 (0.1) |
| Stomach Virus | 1 (0.1) | 0 (0) |
| Strained arm/elbow | 1 (0.1) | 0 (0) |
| Strained back muscles | 1 (0.1) | 0 (0) |
| Strange Dreams | 1 (0.1) | 1 (0.1) |
| Strep throat | 1 (0.1) | 1 (0.1) |
| Strep Throat | 1 (0.1) | 0 (0) |
| Subject had flu | 1 (0.1) | 0 (0) |
| Suicidal thoughts | 1 (0.1) | 1 (0.1) |
| suicide attempt | 1 (0.1) | 1 (0.1) |
| sweating | 6 (0.6) | 6 (0.7) |
| Sweating | 4 (0.4) | 4 (0.5) |
| Sweaty hands | 1 (0.1) | 1 (0.1) |
| swollen hands | 1 (0.1) | 0 (0) |
| Symptoms of aniexty | 1 (0.1) | 1 (0.1) |
| Tachycardia | 1 (0.1) | 1 (0.1) |
| Teeth Grinding | 1 (0.1) | 1 (0.1) |
| Thirsty | 1 (0.1) | 1 (0.1) |
| thoughts about death | 1 (0.1) | 1 (0.1) |
| Throat infection | 1 (0.1) | 0 (0) |
| tight jaw | 1 (0.1) | 1 (0.1) |
| tightness/pressure in head | 1 (0.1) | 1 (0.1) |
| Tingling sensation | 1 (0.1) | 1 (0.1) |
| Tinnitus | 2 (0.2) | 1 (0.1) |
| tired | 2 (0.2) | 2 (0.2) |
| tired, unmotivated | 1 (0.1) | 1 (0.1) |
| tiredness | 3 (0.3) | 2 (0.2) |
| Tiredness | 5 (0.5) | 4 (0.5) |
| Tonsillitis | 1 (0.1) | 0 (0) |
| Tooth ache | 1 (0.1) | 0 (0) |
| Tooth Pain | 1 (0.1) | 0 (0) |
| Toothache | 1 (0.1) | 0 (0) |
| Trembling | 1 (0.1) | 1 (0.1) |
| Tremor | 2 (0.2) | 2 (0.2) |
| tremor feeling | 1 (0.1) | 1 (0.1) |
| trouble sleeping | 1 (0.1) | 1 (0.1) |
| Upper Respiration Infection | 1 (0.1) | 0 (0) |
| upset stomach -mild | 1 (0.1) | 1 (0.1) |
| upset stomach | 3 (0.3) | 3 (0.4) |
| Upset stomach | 2 (0.2) | 2 (0.2) |
| Upset Stomach | 1 (0.1) | 1 (0.1) |
| Uri0 (0)ry Tract Infection | 1 (0.1) | 0 (0) |
| uri0 (0)ting | 1 (0.1) | 1 (0.1) |
| UTI | 1 (0.1) | 0 (0) |
| Virus (head/body aches) | 1 (0.1) | 0 (0) |
| vision impairment | 1 (0.1) | 1 (0.1) |
| vivid dreams | 1 (0.1) | 1 (0.1) |
| Vivid dreams | 1 (0.1) | 1 (0.1) |
| Vivid Nightmare | 1 (0.1) | 1 (0.1) |
| vomiting | 1 (0.1) | 1 (0.1) |
| Vomiting | 2 (0.2) | 1 (0.1) |
| Vomitting | 1 (0.1) | 1 (0.1) |
| wants to take more profit of her good feeling | 1 (0.1) | 1 (0.1) |
| weight gain - approx 2 lbs | 1 (0.1) | 1 (0.1) |
| weight gain | 1 (0.1) | 0 (0) |
| weight loss | 1 (0.1) | 1 (0.1) |
| wisdom tooth pain | 1 (0.1) | 0 (0) |
| Wisdom toothache | 1 (0.1) | 0 (0) |
| Worsening depression | 1 (0.1) | 1 (0.1) |
| worsening difficulties getting out of bed | 1 (0.1) | 1 (0.1) |
| worsening mood | 1 (0.1) | 0 (0) |
| worsening of hip pain | 1 (0.1) | 0 (0) |
| worsening restlesness | 1 (0.1) | 0 (0) |
| wound in foot | 1 (0.1) | 0 (0) |
| Yawning | 2 (0.2) | 2 (0.2) |

**Frequency, Intensity, and Burden of Side Effects**

In the timepoint model, frequency of side effects increased with dosage (*b* = .003, *t* = 3.22, *df* = 643, *p* = .001) and were higher at each timepoint following day 4 (each timepoint *t* > 5 and p < .001). Intensity of side effects increased with depression severity (*b* = .022, *t* = 2.24, *df* = 639, *p* = .026), dosage (*b* = .004, *t* = 4.13, *df* = 628, *p* < .001), and was higher at each timepoint following day 4 (each timepoint *t* > 5 and p < .001). Finally, burden of side effects increased with depression severity (*b* = .027, *t* = 3.15, *df* = 636, *p* = .002), dosage (*b* = .003, *t* = 3.36, df = 626, *p* = .001), and was higher at each timepoint following day 4 (each timepoint *t* > 5 and p < .001).
